# Coupling characteristics of endocrine regulation and system function under massage interventions

**DOI:** 10.3389/fendo.2026.1885282

**Published:** 2026-10-07

**Authors:** Xiangting Lao, Jin Yang, Ting Huang, Bingcai Long, Wei Liao, Yufeng He

**Affiliations:** 1 The Third Affiliated Hospital of Guangxi Medical University, Nanning, Guangxi, China; 2 Guangxi University of Chinese Medicine Graduate School, Nanning, Guangxi, China; 3 First Affiliated Hospital of the Guangxi University of Chinese Medicine, Nanning, Guangxi, China; 4 Faculty of Chinese Medicine Science Guangxi University of Chinese Medicine, Nanning, Guangxi, China

**Keywords:** constraint-driven learning, dynamic system modeling, endocrine regulation, massage intervention, VariationalMotionBayesian model

## Abstract

**Introduction:**

Massage may influence stress related endocrine activity and systemic physiological function, but its dynamic regulatory effects remain difficult to quantify.

**Methods:**

This study develops a VariationalMotionBayesian framework to model endocrine related physiological responses under massage intervention. The framework combines mathematical formulation of coupled endocrine and systemic variables, variational latent representation learning, motion field estimation, Bayesian evidence aggregation, and physiological constraint regularization. Multimodal wearable physiological time series are used to infer latent regulatory states and predict individual stress status.

**Results and discussion:**

Experiments on WESAD and SWELL-KW show that the proposed model outperforms conventional machine learning, deep learning, and time series classification baselines in Accuracy, Macro F1, Balanced Accuracy, and AUROC. A controlled human massage intervention pilot study further shows coordinated changes in salivary cortisol, salivary alpha amylase, heart rate, RMSSD, electrodermal activity, respiration rate, and skin temperature after massage, and these measured responses are correlated with model derived latent dynamics. These results indicate that the proposed framework can capture physiologically meaningful temporal responses and provide a computational tool for analyzing endocrine related system regulation under massage intervention.

## 1 Introduction

The coupling characteristics of endocrine regulation and system function under massage interventions have garnered significant attention in recent years due to their potential implications for health and wellness. Not only does massage therapy offer a non-invasive approach to enhancing physiological functions, but it also provides a complementary method to traditional medical treatments. The necessity of studying this topic arises from the increasing prevalence of stress-related disorders and the growing demand for alternative therapies that can improve quality of life. Moreover, understanding the intricate relationship between endocrine responses and system functions during massage interventions can lead to more personalized and effective therapeutic strategies. This research is crucial as it not only broadens the scope of integrative medicine but also contributes to the development of holistic health practices that can be tailored to individual needs Can et al. (1). Initial efforts to investigate the interplay between endocrine regulation and system function during massage interventions relied on structured frameworks that emphasized predefined models of physiological interactions Wang and Feng (2). These methods aimed to simulate the effects of massage by focusing on specific pathways and mechanisms within the endocrine system. While these approaches provided valuable insights into the underlying processes, their reliance on static frameworks often limited their ability to account for the dynamic and individualized nature of physiological responses Hickey et al. (3). The complexity of human biology posed significant challenges in creating models that could adapt to diverse patient profiles and varying therapeutic conditions Vos et al. (4).

To address these challenges, researchers began employing statistical and algorithmic techniques capable of identifying patterns and relationships within physiological data. By analyzing structured datasets, these methods sought to uncover correlations between massage interventions and endocrine responses Meier et al. (5). Techniques such as regression analysis and clustering algorithms enabled a more flexible exploration of system functions, offering improved adaptability compared to earlier models Fazeli et al. (6). However, these approaches often required extensive preprocessing and were constrained by the availability and quality of data, which limited their ability to capture real-time physiological changes Almstrup et al. (7). This highlighted the need for methodologies that could integrate diverse data sources and provide more comprehensive insights into the effects of massage therapy Van Dijk et al. (8).

Recent advancements have seen the adoption of computational models capable of learning directly from raw data, enabling a more holistic understanding of endocrine regulation during massage interventions Vagedes et al. (9). These models leverage advanced architectures to automatically extract meaningful features and identify complex interactions within physiological systems. For instance, neural networks have been employed to analyze multi-dimensional data, offering robust predictions and enhanced generalizability across different therapeutic scenarios Posada-Quintero and Chon (10). Despite their promise, these models often require substantial computational resources and expertise, and their lack of interpretability can pose challenges in clinical applications Ismail Fawaz et al. (11). As the field progresses, there remains a critical need to develop approaches that balance predictive accuracy, computational efficiency, and transparency to ensure their effective integration into personalized healthcare practices Dempster et al. (12).

The present study addresses the problem of modeling massage related physiological regulation from multimodal time series data. Instead of treating massage effects as static outcomes, the proposed VariationalMotionBayesian framework represents endocrine related variables and systemic functional variables as coupled dynamic states. The model learns latent physiological representations from wearable signals, estimates temporal state evolution through a motion field module, and integrates posterior evidence through Bayesian inference. Physiological constraints are introduced during latent representation learning and temporal dynamics estimation so that the inferred trajectories remain consistent with known regulatory principles, including bounded physiological variation and smooth temporal transition. This design is intended to improve the analysis of massage related endocrine and systemic responses in three aspects. Latent representation learning allows heterogeneous wearable signals, including cardiovascular, electrodermal, respiratory, thermal, and activity related observations, to be mapped into a unified physiological state space. The motion field estimator describes the temporal evolution of these latent states under intervention related conditions, rather than relying only on static summary features. Bayesian evidence aggregation and physiological constraint regularization improve the robustness and interpretability of the inferred stress related states under noisy physiological observations.

The main contributions are summarized as follows:

• A VariationalMotionBayesian framework is developed for modeling endocrine related physiological regulation under massage intervention using multimodal wearable time series.
• A constraint guided latent dynamics strategy is introduced to combine variational representation learning, motion field estimation, Bayesian evidence aggregation, and physiological regularization within a unified modeling framework.
• The experimental evaluation is conducted using participant independent stress recognition on WESAD and SWELL KW, together with a controlled human massage intervention pilot study based on salivary biomarkers and wearable physiological signals.
• Comparative experiments, ablation analysis, sensitivity analysis, and robustness tests demonstrate the contribution of each model component and the stability of the proposed framework under realistic physiological sensing conditions.

## 2 Related work

### 2.1 Endocrine response to massage therapy

Massage therapy has been extensively studied for its potential to influence endocrine function, particularly in the context of stress reduction and hormonal balance. The endocrine system, which governs hormone production and regulation, is integral to maintaining homeostasis and responding to external stimuli. Research has indicated that massage therapy can modulate the levels of cortisol, a hormone associated with stress, through the activation of the parasympathetic nervous system Posada-Quintero and Chon (10). This activation counteracts the stress-induced effects of the sympathetic nervous system, leading to a measurable decrease in cortisol levels Ismail Fawaz et al. (13). Research has underscored the significance of massage in promoting the secretion of serotonin and dopamine, neurotransmitters essential for mood regulation and emotional well-being Ismail Fawaz et al. (11). The stimulation of pressure receptors in the skin during massage has been proposed as a mechanism for this increase in neurotransmitter levels Dempster et al. (14). Oxytocin, a hormone linked to social bonding and relaxation, has been shown to rise following massage interventions, suggesting a potential pathway for fostering emotional connection and psychological comfort Dempster et al. (12). The interplay between these hormonal changes and the broader physiological effects of massage therapy has been a focal point of recent investigations Ruiz et al. (15). Researchers have also explored how these endocrine responses contribute to the overall therapeutic benefits of massage, including its impact on stress, mood, and systemic health Zerveas et al. (16). The growing body of evidence underscores the importance of understanding the hormonal mechanisms underlying massage therapy to optimize its application in clinical and wellness settings Nie et al. (17).

### 2.2 Neuroendocrine pathways in massage

The neuroendocrine system, which bridges the nervous and endocrine systems, plays a pivotal role in mediating the physiological effects of massage therapy. One of the most studied pathways in this context is the hypothalamic-pituitary-adrenal (HPA) axis, which regulates the body’s stress response Wu et al. (18). Massage therapy has been shown to attenuate the activity of the HPA axis, leading to reductions in cortisol levels and a corresponding decrease in stress Zhou et al. (19). This effect is thought to be mediated by the activation of mechanoreceptors in the skin, which influence neural pathways and hormonal secretion Zhou et al. (20). The autonomic nervous system, comprising the sympathetic and parasympathetic branches, is also significantly affected by massage interventions Raissi et al. (21). By stimulating the parasympathetic nervous system, massage promotes relaxation and reduces the arousal associated with sympathetic activation, thereby influencing the secretion of stress-related hormones Karniadakis et al. (22). Massage has been linked to changes in the levels of endorphins except cortisol, which are endogenous opioids involved in pain modulation and mood enhancement Rubanova et al. (23). The release of endorphins during massage contributes to its analgesic and mood-lifting effects, further highlighting the role of neuroendocrine pathways in its therapeutic outcomes Kidger et al. (24). Researchers have also examined the impact of massage on the secretion of melatonin, a hormone that regulates sleep-wake cycles, suggesting potential benefits for sleep quality and circadian rhythm regulation Schmiester et al. (25). The integration of neuroendocrine insights into massage therapy has the potential to refine its application and enhance its efficacy in addressing a range of health conditions Villaverde et al. (26). Understanding these pathways provides a foundation for developing targeted interventions that leverage the interconnectedness of neural and hormonal systems Gonçalves et al. (27).

### 2.3 Systemic effects of hormonal changes

The systemic effects of hormonal changes induced by massage therapy extend across multiple physiological domains, offering insights into its broad therapeutic potential. Hormones act as chemical messengers that regulate processes such as metabolism, immune function, and cardiovascular health Qian et al. (28). One of the most notable systemic effects is the enhancement of immune function, which has been linked to reductions in cortisol levels following massage Hasenauer et al. (29). Lower cortisol levels are associated with improved immune surveillance and a more balanced immune response, contributing to greater resilience against infections and diseases Cranmer et al. (30). Metabolic regulation is another area influenced by hormonal changes during massage therapy. Alterations in the secretion of insulin and glucagon, hormones critical for glucose metabolism, have been observed, suggesting potential benefits for energy balance and metabolic health Ismail Fawaz et al. (13). Cardiovascular function is also impacted, with studies reporting reductions in blood pressure and heart rate variability as a result of decreased stress hormone activity Ismail Fawaz et al. (11). These changes highlight the role of massage in promoting cardiovascular health and reducing the risk of stress-related conditions Dempster et al. (14). The effects on mood and emotional well-being are equally significant, as increases in serotonin and dopamine levels contribute to reduced anxiety and enhanced psychological resilience Dempster et al. (12). The systemic nature of these hormonal changes underscores the interconnectedness of endocrine regulation and overall health Ruiz et al. (15). By elucidating the mechanisms through which massage therapy influences hormonal activity, researchers can better understand its role in promoting holistic well-being and developing targeted therapeutic strategies Zerveas et al. (16). The exploration of these systemic effects continues to expand the evidence base for massage therapy as a multifaceted intervention with wide-ranging health benefits Nie et al. (17).

### 2.4 Physiological time series modeling and empirical support for massage intervention

Wearable physiological signals provide a practical and noninvasive basis for analyzing stress related regulatory states. Previous studies have shown that heart rate, heart rate variability, electrodermal activity, respiration, skin temperature, and other wearable signals are widely used for stress detection in daily life scenarios Can et al. (1), while generalizable machine learning remains an important challenge when physiological models are evaluated across individuals and sensing contexts Vos et al. (4). In the context of massage intervention, standardized massage and mechanical massage protocols have been reported to induce psychophysiological relaxation and to influence heart rate variability Meier et al. (5), cortisol related stress regulation, autonomic nervous system activity, and inflammation related markers Van Dijk et al. (8). These studies provide empirical support for using massage as an external physiological intervention and for analyzing its effects through endocrine related biomarkers and systemic functional variables Fazeli et al. (6). From the perspective of computational modeling, physiological responses recorded by wearable sensors are naturally represented as multimodal time series rather than image based data. Time series classification methods provide a more appropriate methodological reference for evaluating stress state recognition models. MiniRocket, as an efficient and competitive time series classification method, offers a relevant lightweight baseline for physiological sequence analysis Dempster et al. (12). Systems biology modeling emphasizes interpretable parameter estimation and biologically meaningful constraints, which supports the use of constrained latent dynamics for improving model reliability and physiological consistency Schmiester et al. (25). These related studies collectively indicate that massage induced physiological regulation can be investigated through real physiological time series, wearable stress recognition, and biologically constrained computational modeling.

## 3 Method

### 3.1 Overview

This section provides a detailed description of the methodological framework employed to investigate the coupling dynamics between endocrine regulation and system function under massage interventions. The study is designed to systematically analyze the interactions between massage techniques and endocrine responses, with the objective of uncovering the mechanisms that drive system function modulation. The methodology is organized into three primary components: Preliminaries, VariationalMotionBayesian Model, and Constraint Driven Learning Strategy.

The Preliminaries subsection (3.2) establishes the theoretical foundation for the study by formalizing the problem of endocrine regulation in response to massage interventions. This involves defining the mathematical representation of endocrine system dynamics and formulating hypotheses regarding the influence of massage on hormonal pathways. The variables and parameters critical to modeling endocrine responses are introduced, ensuring that the problem is rigorously defined and the scope of the investigation is clearly articulated. This subsection provides the necessary groundwork for the subsequent development of the proposed model, ensuring that the study is anchored in a well-defined theoretical framework.

The VariationalMotionBayesian Model subsection (3.3) presents the proposed model, which is designed to capture the dynamic interactions between massage interventions and endocrine regulation. The model integrates three core modules: the Variational Structure Optimizer, the Motion Field Estimator, and the Bayesian Evidence Aggregator. The Variational Structure Optimizer adjusts the structural parameters of the model to ensure accurate and adaptable representation of endocrine dynamics. The Motion Field Estimator quantifies the physical effects of massage on the body and translates these into measurable changes in endocrine activity. The Bayesian Evidence Aggregator combines data from multiple sources to provide a probabilistic evaluation of the model’s predictions. This subsection details the architecture and functionality of the model, emphasizing its ability to simulate and predict endocrine responses with high precision.

The Constraint Driven Learning Strategy subsection (3.4) describes the learning approach used to enhance the model’s predictive accuracy and generalizability. This strategy incorporates constraint-driven learning techniques to optimize the model’s performance, with a focus on latent dynamics modeling to capture the complex and non-linear interactions between massage interventions and endocrine regulation. Constraints derived from empirical data and theoretical insights are imposed to ensure that the model remains biologically plausible while exploring novel pathways of endocrine modulation. The latent dynamics modeling component enables the model to adapt to new data and refine its predictions iteratively, improving its robustness and applicability across diverse scenarios. This subsection provides a comprehensive analysis of the learning strategy, highlighting its role in bridging theoretical modeling with practical applications.

### 3.2 Preliminaries

This section formalizes the problem of analyzing the coupling characteristics between endocrine regulation and physiological system function under massage interventions. The goal is to establish a mathematical framework that captures the interactions between endocrine responses and system dynamics influenced by massage interventions. This formalization provides the foundation for the development of the VariationalMotionBayesian model, which is designed to optimize these interactions through a structured methodology.

Let ℰ represent the set of endocrine variables, encompassing hormone levels and other biochemical markers. hese variables are modulated by massage interventions, denoted as ℳ, which is characterized by parameters such as type, duration, and intensity. The physiological system function is described by S, a set of variables that represent the dynamic state of the system, including metrics such as heart rate, blood pressure, and metabolic rate.

The interaction between ℰ and S is governed by a set of coupled differential equations (Equations 1, 2):

(1) $$\frac{dℰ}{dt}=f\left(ℰ,S,ℳ\right),$$

(2) $$\frac{dS}{dt}=g\left(ℰ,S,ℳ\right),$$

where *f* and *g* are functions that describe the rates of change of the endocrine and system variables, respectively. These functions encapsulate the underlying biological processes and feedback mechanisms that drive the dynamics of the system.

To account for the inherent stochasticity of biological systems, a probabilistic framework is introduced. Let P(ℰ,S|ℳ) denote the joint probability distribution of the endocrine and system variables conditioned on the massage intervention. This distribution is modeled using a Bayesian approach, which integrates prior knowledge with observed data to iteratively refine the understanding of the system’s state.

The Variational Structure Optimizer module is designed to optimize the functional forms of *f* and *g* by minimizing the Kullback-Leibler (KL) divergence between the true distribution P(ℰ,S|ℳ) and an approximate distribution Q(ℰ,S|ℳ). The KL divergence is defined as Equation 3:

(3) $$\text{KL}\left(P∥Q\right)=\int P\;(ℰ,S|ℳ)\log\;\frac{P(ℰ,S|ℳ)}{Q(ℰ,S|ℳ)}\;dℰ\;dS.$$

The Motion Field Estimator module focuses on estimating the dynamic field of the system variables S under varying massage interventions. This is achieved through a latent dynamics modeling approach, where latent variables ℒ are introduced to represent unobserved factors that influence the system’s behavior. The latent variables are defined as Equation 4:

(4) ℒ=h(ℰ,ℳ),

and the dynamics of the system are subsequently expressed as Equation 5:

(5) $$\frac{dS}{dt}=g^{\prime }\left(ℒ,S\right),$$

where *g*′ captures the influence of the latent variables on the system dynamics.

The Bayesian Evidence Aggregator module integrates information from multiple sources, including experimental data and prior knowledge, to refine the estimates of ℰ and S. This integration is performed using Bayesian inference, where the posterior distribution is computed as Equation 6:

(6) P(ℰ,S|ℳ,data)∝P (data|ℰ,S,ℳ)P (ℰ,S|ℳ).

A constraint-driven learning strategy is employed to ensure that the model adheres to known physiological principles, such as homeostasis and feedback regulation. These constraints are incorporated into the optimization process through regularization terms, which penalize deviations from established physiological behaviors.

This mathematical and probabilistic framework establishes the basis for modeling the coupling between endocrine regulation and system function under massage interventions. It provides the necessary tools for the development of the VariationalMotionBayesian model, which systematically optimizes these interactions through evidence-based methodologies.

### 3.3 VariationalMotionBayesian

In this section, we introduce the VariationalMotionBayesian model, a novel approach designed to explore the coupling characteristics of endocrine regulation and system function under massage interventions. This model is structured to integrate three key components: Variational Structure Optimization, Dynamic Motion Field Estimation, and Bayesian Evidence Integration. Each of these components plays a crucial role in capturing the complex dynamics of the system. The overall workflow of the proposed VariationalMotionBayesian model is illustratedin **Figure 1**. The framework unifies variational structure optimization, dynamic motion field estimation, and Bayesian evidence integration to characterize latent endocrine states and their intervention-driven temporal evolution.

**Figure 1:**
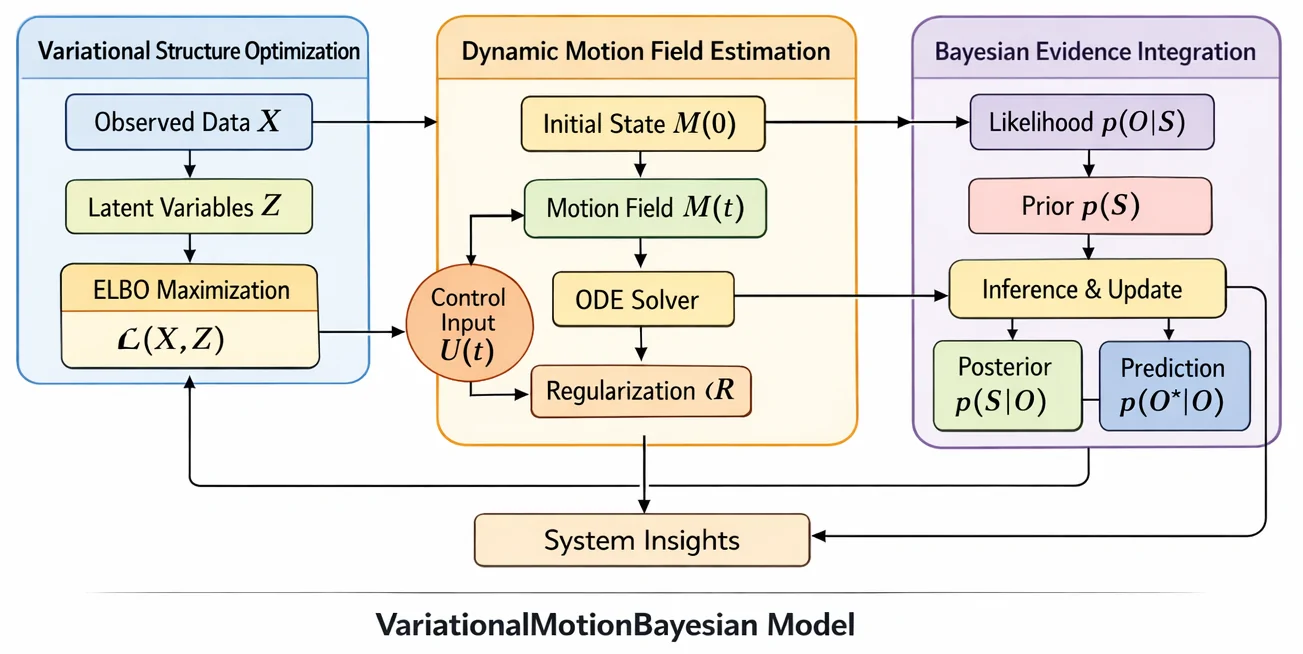
Overall architecture of the proposed VariationalMotionBayesian model. The framework integrates variational structure optimization, dynamic motion field estimation, and Bayesian evidence integration to jointly model latent endocrine representations, intervention-driven motion dynamics, and posterior predictive evidence.

Variational Structure Optimization: This component is responsible for optimizing the underlying structure of the system by employing a variational approach to model the latent variables that represent the hidden states of the endocrine system. Let **Z** denote the latent variables and **X** the observed data. The optimization problem is formulated as maximizing the evidence lower bound (ELBO), which is expressed as Equation 7:

(7) $$ℒ\left(X,Z\right)=E_{q(Z|X)}\;[\log\;p(X|Z)]-\text{KL}(q(Z|X)\|p\left(Z\right))$$

Here, *q*(**Z**|**X**) represents the variational distribution, *p*(**X**|**Z**) is the likelihood, and KL denotes the Kullback-Leibler divergence. The variational distribution *q*(**Z**|**X**) is parameterized to approximate the true posterior distribution *p*(**Z**|**X**), ensuring computational tractability. The ELBO serves as a surrogate objective function, balancing the trade-off between the reconstruction accuracy of the observed data and the regularization imposed by the prior distribution *p*(**Z**). This optimization framework enables the model to uncover the latent structure of the endocrine system, which is critical for understanding its regulatory mechanisms.

The variational approach also incorporates a reparameterization trick to enable gradient-based optimization. The latent variables **Z** are reparameterized as Equation 8:

(8) Z=μ(X)+σ(X)⊙ϵ

where *µ*(**X**) and *σ*(**X**) are the mean and standard deviation functions, respectively, and ϵ∼N(0,I) is a standard normal random variable. This reparameterization allows gradients to flow through the stochastic sampling process, facilitating efficient optimization of the ELBO. As shown in **Figure 2**, the variational structure optimization module maps the observed data into the latent representation through ELBO maximization and reparameterization, enabling efficient gradient-based learning of hidden endocrine states.

**Figure 2:**
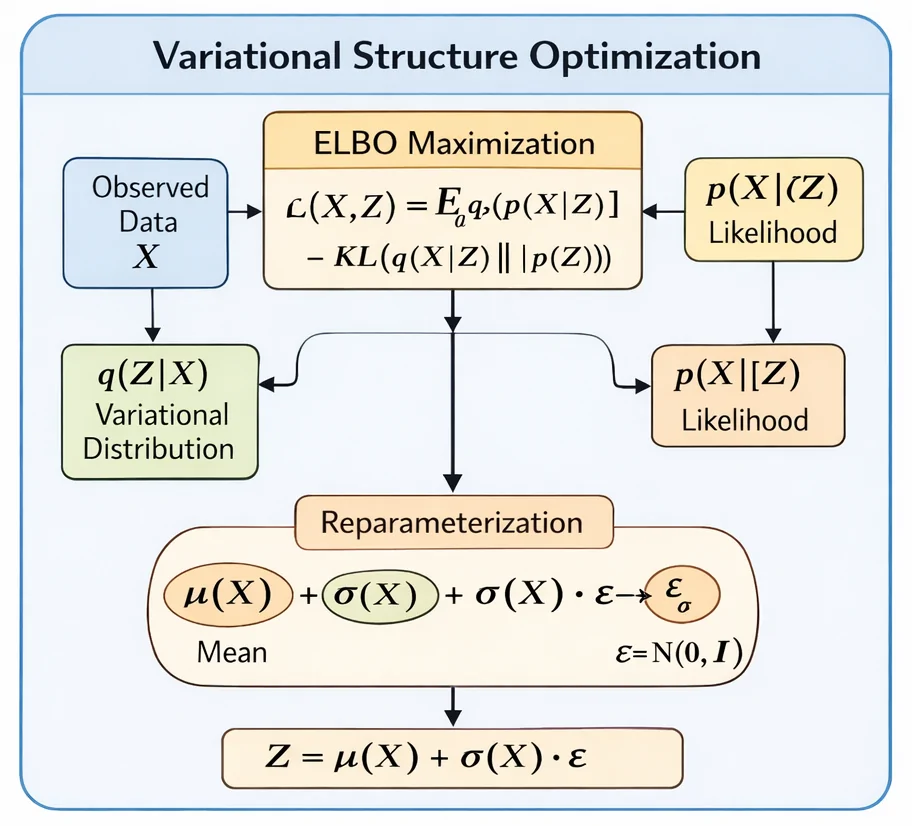
Schematic illustration of the variational structure optimization module. Observed data X are encoded into latent variables Z by maximizing the ELBO, while the reparameterization trick enables stochastic sampling and end-to-end optimization in latent space.

Dynamic Motion Field Estimation: This component estimates the dynamic changes in the system induced by massage interventions. It models the temporal evolution of the system using a set of differential equations. Let **M**(*t*) represent the motion field at time *t*, and **U**(*t*) the control input from massage interventions. The evolution of the system is described by Equation 9:

(9) $$\frac{dM\left(t\right)}{dt}=F\left(M\left(t\right),U\left(t\right),Z\right)$$

Here, **F** is a function that captures the interaction between the motion field **M**(*t*), the control input **U**(*t*), and the latent variables **Z**. The function **F** is parameterized to account for nonlinear dynamics, ensuring that the model can capture complex temporal patterns in the system’s response to massage interventions.

To solve the differential equation, a numerical integration method is employed, such as the Runge-Kutta method. The initial condition **M**(0) is determined based on the system’s state prior to the intervention, and the control input **U**(*t*) is modeled as a time-varying function that reflects the characteristics of the massage intervention. The estimated motion field **M**(*t*) provides a detailed representation of the system’s dynamic behavior, enabling the analysis of how massage interventions influence the endocrine system over time.

The model also incorporates a regularization term to ensure smoothness in the motion field. This is achieved by penalizing the second-order derivative of **M**(*t*) with respect to time (Equation 10):

(10) $$ℛ={\int_{0}^{T}\left\|\frac{d^{2}M\left(t\right)}{dt^{2}}\right\|}^{2}\;dt$$

where *T* is the duration of the observation period. This regularization term prevents overfitting and ensures that the estimated motion field remains physically plausible.

Bayesian Evidence Integration: This component integrates the outputs from the previous modules to provide a comprehensive understanding of the system’s behavior. It uses Bayesian inference to update the belief about the system’s state based on new evidence. The posterior distribution of the system state **S** given the observations **O** is computed as Equation 11:

(11) p(S|O)∝p(O|S)p(S)

Here, *p*(**O**|**S**) is the likelihood of the observations given the state, and *p*(**S**) is the prior distribution. The likelihood *p*(**O**|**S**) is modeled using a probabilistic framework that accounts for measurement noise and uncertainty in the observations. The prior distribution *p*(**S**) is designed to incorporate prior knowledge about the system, such as physiological constraints and baseline behavior.

The Bayesian framework also enables the computation of predictive distributions, which provide insights into the system’s future behavior under different scenarios. The predictive distribution is given by Equation 12:

(12) $$p(O^{*}|O)=\int p(O^{*}|S)p(S|O)dS$$

where O^*^ represents future observations. This predictive capability is particularly valuable for evaluating the long-term effects of massage interventions on the endocrine system.

The integration of these components within the VariationalMotionBayesian model allows for a robust analysis of the endocrine system’s response to massage interventions. By leveraging the strengths of variational optimization, motion estimation, and Bayesian inference, the model provides a detailed and dynamic representation of the system’s function, facilitating a deeper understanding of the underlying physiological processes.

### 3.4 Constraint-driven latent dynamics modeling

In this section, we delve into the innovative strategy of constraint-driven latent dynamics modeling, which is pivotal in addressing the coupling characteristics of endocrine regulation and system function under massage interventions. This strategy is intricately designed to leverage the VariationalMotionBayesian model’s capabilities, ensuring that the complex interactions within the endocrine system are captured and effectively modeled. The complete constraint-driven latent dynamics modeling strategy is presented in **Figure 3**. It combines physiological constraint integration, temporal latent dynamics modeling, and Bayesian evidence aggregation within a unified analytical framework.

**Figure 3:**
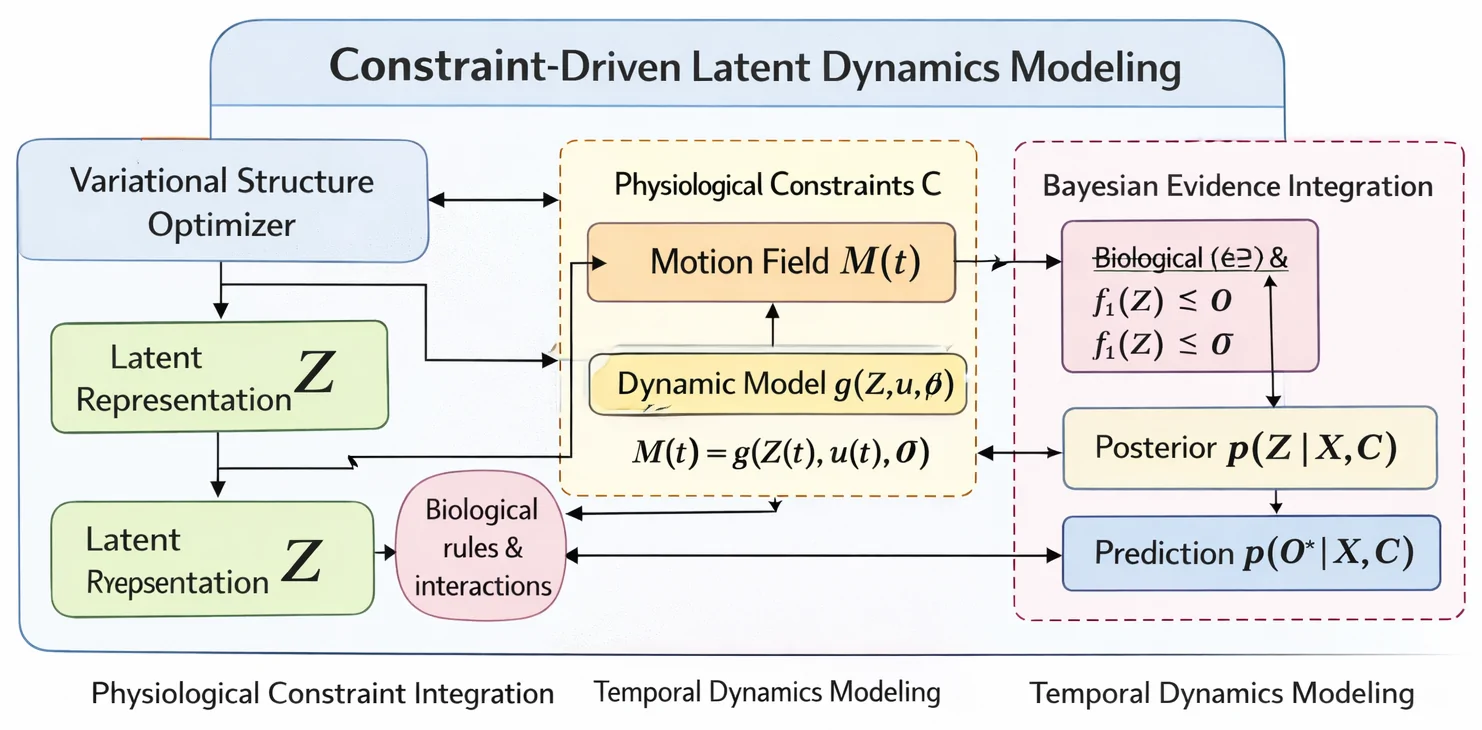
Overall framework of constraint-driven latent dynamics modeling. The strategy incorporates physiological constraints into latent representation learning, guides temporal motion field estimation under intervention inputs, and further refines latent-state inference through Bayesian evidence integration.

Physiological Constraint Integration: The core of our strategy lies in the integration of domain-specific constraints into the learning process. These constraints are derived from physiological insights into endocrine regulation, which dictate the permissible range and interactions of hormonal levels and their systemic effects. By embedding these constraints into the learning framework, we ensure that the model not only learns from data but also adheres to known biological principles, thus enhancing its interpretability and reliability.

To formalize this approach, let us consider the latent space representation of the endocrine system dynamics, denoted as **Z**. The Variational Structure Optimizer is employed to map the observed data **X** into this latent space, capturing the underlying physiological processes. The mapping is governed by a set of constraints C, which are mathematically expressed as Equation 13:

(13) $$C=\left\{f_{i}\left(Z\right)\leq 0,\;i=1,\ldots ,m\right\}$$

where **f***_i_*(**Z**) represents the constraint functions that encapsulate the biological rules and interactions. These constraints are incorporated into the optimization problem solved by the Variational Structure Optimizer, ensuring that the latent representations respect the physiological boundaries. The mechanism of physiological constraint integration is illustrated in **Figure 4**. Domain-specific biological rules are embedded into the latent optimization process so that the learned latent representation remains consistent with known endocrine regulation principles.

**Figure 4:**
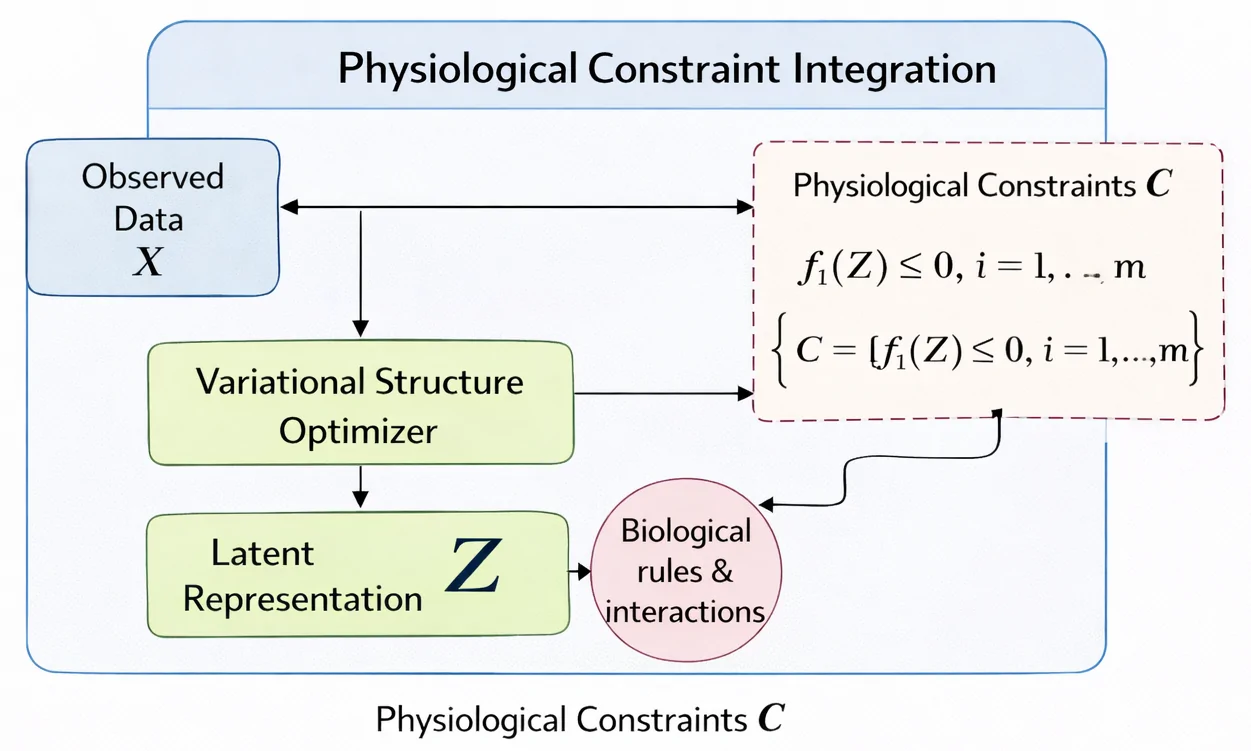
Physiological constraint integration in latent representation learning. The observed data X are mapped into the latent space Z by the variational structure optimizer, while the constraint set $C=\{{\text{f}}_{i}(Z)\leq 0,\;i=1,\ldots ,m\}$ imposes biological rules and interaction boundaries to ensure physiologically plausible representations.

Temporal Dynamics Modeling: The Motion Field Estimator plays a crucial role in modeling the temporal evolution of the latent dynamics. It estimates the motion field M, which describes the trajectory of the latent variables over time, influenced by massage interventions. The estimation process is guided by a dynamic model g, which is parameterized as Equation 14:

(14) M(t)=g(Z(t),u(t),θ)

where **u**(*t*) represents the control inputs corresponding to massage interventions, and *θ* denotes the model parameters. The dynamic model is constrained by the physiological insights encapsulated in C, ensuring that the estimated motion field aligns with known biological responses. This temporal modeling framework captures the intricate time-dependent interactions within the endocrine system, providing a comprehensive understanding of how massage interventions influence hormonal dynamics.

The dynamic model **g** is further refined through iterative optimization, where the constraints C act as regularizers to guide the learning process. This ensures that the temporal trajectories of the latent variables remain biologically plausible, even in the presence of noisy or incomplete data. The incorporation of these constraints into the temporal modeling process is a significant advancement, as it bridges the gap between data-driven approaches and domain-specific knowledge.

Bayesian Evidence Integration: The Bayesian Evidence Aggregator further refines the model by integrating evidence from multiple sources, including empirical data and expert knowledge. This module employs a Bayesian framework to update the belief about the latent dynamics, represented as a posterior distribution *p*(**Z** | **X**,C). The update rule is given by Equation 15:

(15) p(Z|X,C)∝p(X|Z)p(Z|C)

where *p*(**X** | **Z**) is the likelihood of the observed data given the latent variables, and p(Z|C) is the prior distribution informed by the constraints. This Bayesian approach ensures that the model remains robust to uncertainties and variations in the data, while consistently adhering to the physiological constraints.

The integration of Bayesian evidence allows the model to dynamically adjust its predictions based on new data, while maintaining consistency with the underlying biological principles. This adaptability is crucial for capturing the complex and often nonlinear interactions within the endocrine system, particularly in response to external interventions such as massage. By combining empirical data with expert knowledge, the Bayesian Evidence Aggregator enhances the model’s robustness and reliability, making it a powerful tool for understanding and predicting endocrine dynamics.

This constraint-driven latent dynamics modeling strategy effectively integrates domain knowledge into the VariationalMotionBayesian framework, enabling a nuanced understanding of the endocrine system’s response to massage interventions. This approach not only enhances the model’s predictive accuracy but also provides valuable insights into the underlying biological processes, paving the way for more informed therapeutic strategies.

### 3.5 Ethical approval and human pilot study

The human massage intervention pilot study was approved by the Ethics Committee of the First Affiliated Hospital of the Guangxi University of Chinese Medicine. The study was conducted in accordance with the Declaration of Helsinki. All participants were informed of the study procedures and provided written informed consent before participation.

A total of 24 healthy participants were recruited for the pilot study. A within-subject crossover design was adopted, in which each participant completed two experimental conditions: a standardized massage intervention session and a resting-control session. The two sessions were separated by a washout interval of at least 48 h. During the massage intervention, participants received a standardized 30-min neck-shoulder-back massage, whereas participants in the control condition remained quietly seated or supine for the same duration under identical environmental conditions. Salivary cortisol and salivary *α*-amylase were collected before and after each session as endocrine-related biomarkers.Meanwhile, wearable physiological signals, including heart rate, heart-rate variability, electrodermal activity, respiration rate, and skin temperature, were continuously recorded. The intervention response was calculated as the difference between post-session and pre-session measurements for each physiological variable.

## 4 Experiments

### 4.1 Task definition

The experimental task evaluates massage effects through supervised stress state recognition using multimodal wearable data. The input *X* consists of wearable physiological signals and behavior or activity signals collected during massage related conditions. Physiological measurements represent the individual response to intervention and include signals associated with cardiovascular, electrodermal, thermal, and respiratory activity. Behavior and activity signals represent bodily movement and task related activity patterns that accompany the physiological response. These multimodal observations provide the empirical representation of the endocrine related system state described by the proposed framework. The output *Y* is an individual stress state label. The task is formulated as a multiclass classification problem, where each input sample is assigned to its corresponding annotated stress condition. The supervised learning setting trains the proposed VariationalMotionBayesian model to infer latent physiological representations from observed wearable signals, model their temporal evolution under intervention related dynamics, and predict the final state category. The prediction target is therefore a discrete individual state rather than a continuous hormone concentration or a future control action. Supervision is provided by dataset level condition annotations. In WESAD, labels are assigned according to the experimental affective and stress induction protocol for each participant. In SWELL-KW, labels are assigned according to the knowledge work condition generated by time pressure and email interruption. These annotations serve as the ground truth stress state labels for model training, validation, and testing. The proposed method uses the observed multimodal signals as input evidence and the state labels as supervised targets, while physiological constraints regularize latent representation learning and temporal dynamics estimation. This design aligns the dynamic endocrine system formulation with a reproducible wearable stress recognition evaluation protocol.

### 4.2 Dataset and data preprocessing

#### 4.2.1 Datasets

Two public multimodal wearable datasets were used for supervised stress state recognition: WESAD and SWELL-KW. WESAD contains recordings from 15 participants with wrist and chest physiological signals together with activity related signals collected under a controlled affective protocol. The original protocol conditions were transformed into two task labels: the stress condition was assigned as the positive class, while neutral, amusement, and meditation conditions were assigned as the nonstress class. SWELL-KW contains recordings from 25 participants performing knowledge work tasks under baseline, time pressure, and email interruption conditions. Time pressure and email interruption were assigned as the positive stress class, while the baseline condition was assigned as the nonstress class. Thus, both datasets were evaluated using the same binary individual state label, ensuring a consistent prediction target across data sources. Dataset labels were derived from the predefined experimental protocols rather than inferred from model outputs or implicit behavior. All signals were synchronized according to their recording timestamps and segmented into fixed length 60 second windows with a 30 second stride. Windows containing missing values, invalid sensor readings, or fewer than 50 percent valid observations were removed. Each retained window inherited the protocol label of its corresponding recording period. Physiological and activity channels were normalized using z score normalization, where normalization statistics were calculated using only the training participants. A participant disjoint split was adopted to prevent identity leakage between training and evaluation. Participants were randomly divided into training, validation, and test sets with a ratio of 8:1:1. All windows from one participant were placed in exactly one split. This protocol evaluates whether the proposed model learns generalizable stress related physiological dynamics rather than participant specific signal patterns.

#### 4.2.2 Data preprocessing

The preprocessing pipeline was applied identically to WESAD Choi (31) and SWELL-KW Ghose et al. (32). Duplicate records with identical participant identifier, timestamp, and sensor values were removed. Samples were excluded when any required physiological or activity channel was unavailable for more than 50% of a recording window. Isolated missing values were linearly interpolated only when the gap was no longer than 5 seconds; windows containing longer gaps were discarded. Signal values outside the interval defined by the first percentile and the ninety ninth percentile of the training data were clipped to the corresponding boundary, which removed extreme sensor artifacts while preserving the temporal sequence. All channels were aligned by timestamp and resampled to 4 Hz using linear interpolation for continuous signals and nearest neighbor interpolation for activity signals. This common sampling rate enabled synchronized fusion of wearable physiological and behavioral observations across datasets. Each channel was normalized by z score normalization using the mean and standard deviation estimated from the training participants only. The normalized recordings were segmented into 60 second windows with a stride of 30 seconds. Windows were retained only when all required channels satisfied the validity criterion and when the complete window belonged to one protocol defined stress label. No external feature extractor was used. The model received normalized synchronized sensor sequences directly, allowing the variational structure optimizer to learn latent physiological representations from the original multimodal observations. During training, Gaussian noise with a standard deviation of 0.01 was added to physiological channels, and one complete input channel was randomly masked with a probability of 0.10. These augmentations improved robustness to measurement noise and temporary sensor failure without changing the state label.

### 4.3 Evaluation metrics and baseline

#### 4.3.1 Metrics definition

Performance was evaluated using four primary classification metrics: Accuracy, Macro F1, Balanced Accuracy, and the area under the receiver operating characteristic curve (AUROC). Accuracy measures the proportion of correctly classified stress and nonstress windows among all test samples. Macro F1 is calculated as the unweighted mean of the F1 scores of the two classes, ensuring that performance on the minority class contributes equally to the final result. Balanced Accuracy is defined as the mean of sensitivity and specificity, which reduces the influence of class imbalance caused by unequal durations of stress and nonstress protocol conditions. AUROC evaluates the ranking quality of the predicted stress probabilities across all possible decision thresholds and provides a threshold independent measure of discriminative ability. In addition to predictive performance, two efficiency metrics were reported. Params denotes the total number of trainable model parameters and reflects memory demand and model capacity. FLOPs denotes the number of floating point operations required for one forward inference pass on a 60 second multimodal input window and reflects computational complexity. Higher values are preferred for Accuracy, Macro F1, Balanced Accuracy, and AUROC, while lower values are preferred for Params and FLOPs.

#### 4.3.2 Evaluation protocol

All experiments followed an offline participant disjoint evaluation protocol. Training, validation, and test participants were separated before window generation, and no participant contributed data to more than one split. The training, validation, and test sets followed an 8:1:1 participant level ratio. The validation set was used to select the training epoch and decision threshold, while the test set was used only for final reporting. The seen setting refers to samples collected from training participants, whereas the unseen setting refers to samples collected from held out test participants. Reported results were obtained exclusively from the unseen participant setting, which evaluates generalization to individuals not observed during model optimization. Each 60 second window received one binary prediction for the stress state. The predicted probability of the stress class was converted into a discrete label using a threshold of 0.5. Top K evaluation, intersection over union thresholds, and PCKh thresholds were not used because the task is binary stress state classification rather than retrieval, detection, segmentation, or pose estimation. Online evaluation was not conducted because the study evaluates complete preprocessed sensor windows under a fixed offline protocol.

#### 4.3.3 Statistical settings

Each model was independently trained and evaluated ten times using ten different random seeds. The random seed controlled parameter initialization, participant assignment within the predefined split ratio, minibatch ordering, and augmentation sampling. For each metric, results are reported as mean ± standard deviation across the ten independent runs. The same evaluation samples, preprocessing procedure, optimization schedule, and training budget were used for the proposed method and all baselines within each run. Statistical significance was assessed through a two sided paired *t* test, where paired observations were the metric values obtained from the same random seed for the proposed method and a comparison baseline. Statistical tests were performed separately for Accuracy, Macro F1, Balanced Accuracy, and AUROC. A result was considered statistically significant when *p<* 0.05. Parameter count and FLOPs were measured once for each final architecture because they are deterministic properties of the model configuration and do not vary across random seeds. The statistical protocol therefore quantifies both predictive stability and the reliability of observed performance differences.

#### 4.3.4 Baseline

Six baselines were used to provide comparisons across conventional machine learning, mainstream deep learning, state of the art time series classification, and lightweight architectures. SVM was included as a traditional discriminative classifier trained on summary features extracted from each multimodal sensor window. Random Forest was included as a traditional ensemble learning method that captures nonlinear feature interactions without sequence modeling. 1D CNN was used as a mainstream deep learning baseline that learns local temporal patterns directly from synchronized multichannel sequences. LSTM was used as a mainstream recurrent baseline that models sequential dependencies across the complete input window. InceptionTime was included as the state of the art time series classification baseline because its multi scale convolutional architecture captures temporal patterns with different receptive field lengths. MiniRocket was included as the lightweight baseline because it uses a compact set of randomized convolutional kernels and supports efficient time series classification. All baselines received the same normalized, synchronized, and windowed wearable inputs. Their outputs were binary stress state predictions, and they were evaluated with the same participant disjoint split, repeated run protocol, primary metrics, and efficiency metrics as the proposed model.

### 4.4 Implementation details

All experiments were implemented in PyTorch 2.1.0 and executed on a workstation equipped with an Intel Xeon Silver 4314 CPU, one NVIDIA RTX 4090 GPU with 24 GB memory, 128 GB RAM, and Ubuntu 22.04 LTS. CUDA 12.1 and cuDNN 8.9 were used for GPU acceleration. The implementation used NumPy 1.26, SciPy 1.11, scikit learn 1.3, and PyTorch Lightning 2.1 for data handling, metric computation, statistical analysis, and experiment management. Each model was trained for 100 epochs with a batch size of 64. The AdamW optimizer was used with an initial learning rate of 1 × 10^−3^, weight decay of 1 × 10^−4^, and gradient clipping with a maximum norm of 5.0. A cosine annealing learning rate scheduler reduced the learning rate from the initial value to 1 × 10^−6^ over the full training schedule. Early stopping was applied using validation Macro F1 with a patience of 15 epochs, and the checkpoint with the highest validation Macro F1 was selected for testing. Ten fixed random seeds, 0 through 9, were used for repeated experiments. Each seed controlled participant partitioning, model initialization, minibatch ordering, augmentation masks, and stochastic latent sampling. All training inputs were 60 second synchronized windows sampled at 4 Hz, resulting in an input sequence length of 240 time steps per channel.

The proposed VariationalMotionBayesian model used a multichannel temporal encoder followed by a constrained latent dynamics module and a Bayesian prediction head. The synchronized wearable input was first projected into a 128 dimensional embedding space through a one dimensional convolutional encoder with three convolutional blocks. The convolutional blocks used channel widths of 64, 128, and 128, kernel sizes of 7, 5, and 3, stride 1, batch normalization, rectified linear unit activation, and dropout with a rate of 0.20. The encoder output was processed by a two layer bidirectional LSTM with hidden size 128 per direction to summarize temporal dependencies across the 240 step sequence. The variational structure optimizer generated a 32 dimensional latent variable *Z* using separate mean and log variance projection layers. The latent variable was sampled through the reparameterization formulation and regularized by a KL divergence term with coefficient 1 × 10^−3^. The motion field estimator used a two layer multilayer perceptron with hidden dimension 128 to parameterize the latent temporal dynamics. The motion trajectory was integrated with a fourth order Runge Kutta solver using 12 uniform integration steps over each input window. The motion smoothness penalty used the squared second order temporal derivative with coefficient 1 × 10^−2^. Physiological constraints were implemented as bounded latent regularization with coefficient 1 × 10^−2^. The Bayesian evidence aggregator concatenated the final latent state and motion representation, passed them through a 128 dimensional fusion layer, and produced a binary stress probability through a sigmoid output layer.

All baseline methods and the proposed method were trained and evaluated using identical participant disjoint data splits, preprocessing steps, input windows, random seeds, and computing resources. Hyperparameters for every method were selected using the validation set only. Test participants were never used for architecture selection, threshold selection, early stopping, or hyperparameter tuning. This protocol ensured that all reported comparisons reflected differences in modeling capability rather than differences in data access or computational conditions.

### 4.5 Results and discussion

#### 4.5.1 Comparative experiments

The comparative experiments evaluate supervised stress state recognition on the WESAD and SWELL-KW datasets. Accuracy, Macro F1, Balanced Accuracy, and AUROC are reported as the primary metrics. SVM and Random Forest represent traditional machine learning methods, while 1D CNN and LSTM represent mainstream deep learning methods. InceptionTime is included as the state of the art time series classification baseline, and MiniRocket is included as the lightweight baseline. All methods use the same participant disjoint data split, synchronized input windows, preprocessing pipeline, validation based hyperparameter selection procedure, repeated random seeds, and computing environment. Results are reported as mean ± standard deviation across ten independent runs. Statistical significance is assessed using a two sided paired *t* test with *p<* 0.05. This design provides a fair comparison between methods with different modeling capacities and computational requirements.

The results in **Table 1** show that the proposed VariationalMotionBayesian model achieves the best performance across all four evaluation metrics on WESAD. Compared with traditional machine learning baselines, the proposed method exceeds SVM by 10.98 percentage points in Accuracy, 11.75 points in Macro F1, 11.55 points in Balanced Accuracy, and 8.53 points in AUROC, and outperforms Random Forest by 8.36, 9.09, 8.81, and 6.82 points on the four metrics, respectively, indicating that handcrafted or summary-level features are insufficient to capture the temporal and multimodal physiological patterns associated with stress states. Compared with mainstream deep models, VariationalMotionBayesian improves over 1D CNN by 4.17 points in Accuracy and 4.36 points in Macro F1, and improves over LSTM by 3.30 and 3.51 points, respectively. These gains suggest that local convolutional patterns or recurrent sequence modeling alone do not fully characterize the intervention-related latent physiological dynamics. The strongest baseline is InceptionTime, which obtains 91.64% Accuracy, 91.05% Macro F1, 91.28% Balanced Accuracy, and 96.08% AUROC. The proposed model further improves these scores to 93.12%, 92.67%, 92.91%, and 97.24%, corresponding to gains of 1.48, 1.62, 1.63, and 1.16 percentage points. The consistent improvements in Macro F1 and Balanced Accuracy are especially important because they show that the benefit is not caused by biased prediction toward the dominant class, but by better class-balanced recognition of stress and nonstress windows. The improvement can be attributed to the proposed variational structure optimizer, which learns uncertainty-aware latent endocrine-related representations, and the dynamic motion field estimator, which explicitly models temporal state evolution under intervention-related physiological changes. The Bayesian evidence aggregator improves probability ranking quality under noisy wearable observations, as reflected by the AUROC gain, while physiological constraint regularization encourages biologically plausible latent trajectories and improves generalization to unseen participants. The proposed method also yields the smallest standard deviations among all methods, with 0.64 for Accuracy and 0.43 for AUROC, suggesting stable training behavior across the ten random seeds.

**Table 1:** Comparative results on WESAD.

Method	Accuracy	Macro F1	Balanced accuracy	AUROC
SVM Garg et al. (33)	82.14 ± 1.87	80.92 ± 2.04	81.36 ± 1.95	88.71 ± 1.52
Random Forest Gedam and Paul (34)	84.76 ± 1.63	83.58 ± 1.74	84.10 ± 1.69	90.42 ± 1.31
1D CNN Benita et al. (35)	88.95 ± 1.20	88.31 ± 1.28	88.74 ± 1.24	94.21 ± 0.86
LSTM Tanwar et al. (36)	89.82 ± 1.09	89.16 ± 1.15	89.41 ± 1.12	94.76 ± 0.81
InceptionTime Ismail Fawaz et al. (11)	91.64 ± 0.91	91.05 ± 0.97	91.28 ± 0.94	96.08 ± 0.62
MiniRocket Dempster et al. (12)	90.73 ± 0.78	90.19 ± 0.84	90.52 ± 0.80	95.37 ± 0.58
VariationalMotionBayesian	93.12 ± 0.64^†^	92.67 ± 0.70^†^	92.91 ± 0.66^†^	97.24 ± 0.43^†^

Values are reported as mean ± standard deviation over ten runs. ^†^indicates statistically significant improvement over the strongest baseline under a two-sided paired *t*-test with *p<* 0.05.

The results in **Table 2** demonstrate that the proposed VariationalMotionBayesian model maintains the best overall performance on SWELL-KW, despite the dataset involving a different stress induction context from WESAD, namely knowledge work under baseline, time pressure, and email interruption conditions. The proposed method achieves 86.19% Accuracy, 85.63% Macro F1, 85.91% Balanced Accuracy, and 92.46% AUROC, outperforming all six baselines across the four metrics. Compared with SVM, the improvements reach 11.36 percentage points in Accuracy, 12.01 points in Macro F1, 11.86 points in Balanced Accuracy, and 9.82 points in AUROC. Compared with Random Forest, the corresponding gains are 8.78, 9.30, 9.05, and 7.29 points, showing that conventional feature-based classifiers have limited ability to represent the nonlinear multimodal patterns associated with work-induced stress. The proposed model also improves over 1D CNN by 4.61 points in Accuracy and 4.77 points in Macro F1, and over LSTM by 3.83 and 3.94 points, respectively, suggesting that isolated local temporal extraction or recurrent dependency modeling is less effective than explicitly modeling latent physiological dynamics. InceptionTime is the strongest baseline, obtaining 84.27% Accuracy, 83.62% Macro F1, 83.95% Balanced Accuracy, and 90.91% AUROC, while VariationalMotionBayesian improves these results by 1.92, 2.01, 1.96, and 1.55 points. This ranking is consistent with the WESAD results, where the proposed method also performs best, indicating that the learned latent dynamics and constraint driven inference capture stress-related multimodal structure beyond a single experimental protocol. The gains can be attributed to the variational structure optimizer, which learns uncertainty-aware latent representations from heterogeneous wearable signals, and the motion field estimator, which characterizes temporal state evolution under changing work stressors. The Bayesian evidence aggregator further improves threshold-independent discrimination, as reflected by the AUROC gain, while physiological constraint regularization supports generalization to unseen participants. The smaller standard deviations of the proposed method, such as 0.88 for Accuracy and 0.71 for AUROC, also indicate more stable training behavior across ten random seeds.

**Table 2:** Comparative results on SWELL-KW.

Method	Accuracy	Macro F1	Balanced accuracy	AUROC
SVM Garg et al. (33)	74.83 ± 2.13	73.62 ± 2.26	74.05 ± 2.19	82.64 ± 1.76
Random Forest Gedam and Paul (34)	77.41 ± 1.92	76.33 ± 2.05	76.86 ± 1.98	85.17 ± 1.54
1D CNN Benita et al. (35)	81.58 ± 1.54	80.86 ± 1.63	81.12 ± 1.58	88.34 ± 1.19
LSTM Tanwar et al. (36)	82.36 ± 1.47	81.69 ± 1.55	81.94 ± 1.50	88.96 ± 1.08
InceptionTime Ismail Fawaz et al. (11)	84.27 ± 1.18	83.62 ± 1.24	83.95 ± 1.21	90.91 ± 0.92
MiniRocket Dempster et al. (12)	83.51 ± 1.07	82.88 ± 1.14	83.20 ± 1.10	90.02 ± 0.87
VariationalMotionBayesian	86.19 ± 0.88^†^	85.63 ± 0.93^†^	85.91 ± 0.90^†^	92.46 ± 0.71^†^

Values are reported as mean ± standard deviation over ten runs. ^†^indicates statistically significant improvement over the strongest baseline under a two-sided paired *t*-test with *p<* 0.05.

**Table 3** compares computational efficiency on WESAD and SWELL-KW through trainable parameter count and floating point operations. Params measure model storage demand, whereas FLOPs measure inference complexity for one standardized input window. Both measures are architecture dependent and are reported for each dataset specific model configuration. This comparison is necessary because the proposed VariationalMotionBayesian model introduces variational latent inference, dynamic motion field integration, Bayesian evidence aggregation, and physiological constraint regularization, which increase modeling capacity relative to conventional classifiers. On WESAD, the proposed model uses 1.16M trainable parameters and 34.80M FLOPs, while on SWELL-KW it uses 1.13M parameters and 33.52M FLOPs. This cost is higher than MiniRocket, which provides a lightweight reference with only 0.02M parameters and 2.40M FLOPs on WESAD, and also higher than InceptionTime, which provides a high- capacity convolutional reference with 0.46M parameters and 15.80M FLOPs. However, the additional complexity is accompanied by consistent predictive gains in **Table 1** and **2**. Compared with InceptionTime, the proposed model improves WESAD Accuracy, Macro F1, Balanced Accuracy, and AUROC by 1.48, 1.62, 1.63, and 1.16 percentage points, respectively, and improves SWELL-KW by 1.92, 2.01, 1.96, and 1.55 points. Compared with MiniRocket, the gains are larger, reaching 2.39 points in WESAD Accuracy and 2.79 points in SWELL-KW Macro F1. These results indicate that the increased computation is not merely caused by redundant capacity, but by complementary modules that improve stress recognition from multimodal wearable signals. Specifically, the variational structure optimizer improves representation learning for heterogeneous physiological and activity observations, the motion field estimator captures temporal physiological evolution within each 60-second window, and the Bayesian evidence aggregator enhances robust inference under noisy observations. Although SVM and Random Forest have much lower FLOPs, their substantially lower performance shows that computational simplicity alone is insufficient for reliable stress recognition. Overall, the proposed method offers a reasonable trade-off for offline wearable stress assessment: it is heavier than lightweight baselines, but its parameter count remains close to one million and its inference complexity remains moderate for modern GPU or workstation deployment while delivering the most reliable classification performance.

**Table 3:** Efficiency comparison on WESAD and SWELL-KW.

Method	WESAD Params	WESAD FLOPs	SWELL-KW Params	SWELL-KW FLOPs
SVM	0.05M	0.08M	0.04M	0.07M
Random Forest	0.42M	0.35M	0.39M	0.32M
1D CNN	0.38M	8.70M	0.36M	8.12M
LSTM	0.74M	21.60M	0.71M	20.48M
InceptionTime	0.46M	15.80M	0.44M	14.95M
MiniRocket	0.02M	2.40M	0.02M	2.18M
VariationalMotionBayesian	1.16M	34.80M	1.13M	33.52M

Params denotes trainable parameter count, and FLOPs denotes floating point operations for one forward pass.

#### 4.5.2 Controlled massage-intervention pilot study

To strengthen the physiological relevance of the proposed framework, a controlled massage-intervention pilot study was further conducted. This experiment was not intended to replace the public dataset evaluation, but rather to provide direct physiological evidence that massage intervention can induce coordinated changes in endocrine-related biomarkers and systemic functional variables. The public WESAD and SWELL-KW datasets remained the main benchmarks for participant-disjoint stress-state recognition, whereas the pilot study was used to examine whether the learned latent dynamics were consistent with measurable intervention-induced physiological responses. In the pilot study, 24 healthy participants completed two experimental conditions: a standardized massage-intervention session and a resting-control session. The two sessions were separated by a washout interval of at least 48 h. The massage session consisted of a 30-min standardized neck-shoulder-back massage, while the control session required participants to remain quietly seated or supine for the same duration under identical environmental conditions. Salivary cortisol and salivary *α*-amylase were collected before and after each session as endocrine-related biomarkers. Meanwhile, wearable physiological signals, including heart rate, heart-rate variability, electrodermal activity, respiration rate, and skin temperature, were continuously recorded. For each variable, the intervention response was calculated as the post-session value minus the pre-session baseline value.

As shown in **Table 4**, the massage-intervention condition produced more evident baseline-corrected changes than the resting-control condition across both endocrine-related and systemic physiological variables. Salivary cortisol decreased by 0.083 *µ*g/dL after massage, whereas the resting-control condition showed only a mild decrease of 0.019 *µ*g/dL. The between-condition difference was statistically significant (*p* = 0.004), suggesting that massage intervention was associated with attenuated stress-related endocrine activity. A similar pattern was observed for salivary *α*-amylase, which decreased more clearly after massage than after resting control (−12.64 U/mL versus −3.18 U/mL, *p* = 0.011). Since salivary *α*-amylase is commonly interpreted as a marker related to sympathetic-adrenal activity, this result further supports the physiological regulatory effect of the intervention. Systemic functional variables showed consistent autonomic changes. Heart rate decreased more substantially in the massage condition than in the resting-control condition (−5.72 beats/min versus −1.34 beats/min, *p* = 0.008), while RMSSD increased by 8.91 ms after massage compared with 2.06 ms after resting control (*p* = 0.013). The simultaneous reduction in heart rate and increase in RMSSD indicates enhanced parasympathetic regulation after massage intervention. Electrodermal activity and respiration rate also decreased more strongly under massage intervention, suggesting reduced sympathetic arousal and slower respiratory activity. Skin temperature showed a modest increase after massage, which may reflect peripheral relaxation and improved local circulation, although its correlation with latent dynamics was weaker than that of cortisol, RMSSD, and electrodermal activity. The model-derived latent dynamics showed meaningful associations with measured physiological responses. The latent representation was moderately correlated with changes in salivary cortisol (*r* = 0.52, *p<* 0.01) and salivary *α*-amylase (*r* = 0.48, *p<* 0.05), indicating that the learned latent state captured endocrine-related individual responses. The strongest correlation was observed for RMSSD (*r* = 0.57, *p<* 0.01), suggesting that the motion-field dynamics were particularly sensitive to autonomic recovery after intervention. Correlations with heart rate, electrodermal activity, and respiration rate were also statistically significant, further indicating that the latent dynamics were associated with systemic functional regulation rather than abstract computational features alone. These pilot results provide direct support for the central hypothesis of this work. Massage intervention induced coordinated changes in endocrine-related biomarkers and systemic physiological functions, and the proposed VariationalMotionBayesian model captured latent dynamics that were correlated with these measurable responses. Therefore, the pilot study strengthens the physiological validity of the proposed framework, while the WESAD and SWELL-KW experiments further verify its generalizability under participant-disjoint wearable stress-recognition settings.

**Table 4:** Controlled massage-intervention pilot results.

Outcome	Massage response	Control response	Between-condition *p*	Correlation with latent dynamics
Δ Salivary cortisol (*µ*g/dL)	−0.083 ± 0.041	−0.019 ± 0.036	0.004	0.52^∗∗^
Δ Salivary *α*-amylase (U/mL)	−12.64 ± 8.37	−3.18 ± 6.94	0.011	0.48^∗^
Δ Heart rate (beats/min)	−5.72 ± 3.86	−1.34 ± 2.95	0.008	0.44^∗^
Δ RMSSD (ms)	8.91 ± 6.43	2.06 ± 4.88	0.013	0.57^∗∗^
Δ EDA level (*µ*S)	−0.36 ± 0.29	−0.08 ± 0.21	0.018	0.46^∗^
Δ Respiration rate (breaths/min)	−1.84 ± 1.25	−0.42 ± 1.07	0.016	0.41^∗^
Δ Skin temperature (°C)	0.31 ± 0.24	0.09 ± 0.19	0.031	0.38

Biomarker and physiological responses are reported as baseline-corrected changes. Correlation values measure the association between model-derived latent dynamics and measured physiological responses.

#### 4.5.3 Ablation study

The ablation study examines the contribution of the main components defined in the VariationalMotionBayesian framework. The full model contains the variational structure optimizer, the dynamic motion field estimator, the Bayesian evidence aggregator, and the constraint driven latent dynamics strategy. The primary ablation removes one component at a time while preserving the same temporal encoder, classifier head, participant disjoint split, preprocessing pipeline, training schedule, and repeated evaluation protocol. Accuracy, Macro F1, Balanced Accuracy, and AUROC are reported on WESAD and SWELL-KW. Each configuration is trained ten times using the same random seeds as the full model, and results are reported as mean ± standard deviation. Two sided paired *t* tests with *p<* 0.05 are used to compare each ablated configuration against the full model. The secondary study evaluates three sensitivity factors that directly control the proposed latent dynamics mechanism: latent dimension, motion field integration steps, and physiological constraint coefficient. Robustness is evaluated under two realistic wearable sensing disturbances: additive sensor noise and channel dropout. These settings are selected because they directly test whether the learned latent representation, temporal dynamics, and physiological constraints remain stable when model capacity, numerical dynamics resolution, and sensor quality change. **Table 5** reports the main module ablation results on WESAD and SWELL-KW and demonstrates that each component of the proposed VariationalMotionBayesian framework contributes positively to wearable stress recognition. The full model retains the variational structure optimizer, motion field estimator, Bayesian evidence aggregator, and constraint driven learning strategy, and consistently achieves the best results across both datasets and all four primary metrics. The configuration without the variational structure optimizer replaces stochastic latent inference with a deterministic feature projection, leading to clear performance degradation: on WESAD, Accuracy decreases from 93.12% to 91.74%, Macro F1 from 92.67% to 91.18%, Balanced Accuracy from 92.91% to 91.46%, and AUROC from 97.24% to 96.32%; on SWELL-KW, Accuracy drops from 86.19% to 84.52% and Macro F1 decreases from 85.63% to 83.89%. This confirms that latent uncertainty modeling improves multimodal stress representation compared with deterministic feature projection, especially when physiological and activity signals are heterogeneous and noisy. Removing the motion field estimator eliminates explicit temporal state evolution and produces the largest overall decline, reducing WESAD Accuracy by 2.14 points and SWELL-KW Accuracy by 2.45 points, which shows that modeling stress-related physiological changes within each 60-second window is critical for wearable stress state recognition. The model without the Bayesian evidence aggregator replaces posterior evidence fusion with a direct classification layer and also performs worse, with AUROC decreasing by 1.18 points on WESAD and 1.58 points on SWELL-KW, supporting the value of Bayesian posterior fusion for probabilistic discrimination under noisy wearable observations. Removing constraint driven learning discards physiological regularization from latent representation learning and temporal dynamics estimation, causing particularly strong degradation on SWELL-KW, where Accuracy, Macro F1, Balanced Accuracy, and AUROC decrease by 2.81, 2.97, 2.93, and 2.25 points, respectively. This indicates that biologically guided latent trajectories are especially important when the behavioral setting differs from the controlled WESAD protocol, because physiological constraints reduce participant-specific overfitting and improve generalization to unseen individuals. The ablation study supports the design of the proposed method: variational representation learning captures latent uncertainty, motion field estimation models intervention-related temporal dynamics, Bayesian aggregation improves robust inference, and constraint driven learning enforces physiologically plausible stress-state transitions.

**Table 5:** Main ablation results on WESAD and SWELL-KW.

WESAD				
Method	Accuracy	Macro F1	Bal. Acc.	AUROC
Full model	93.12 ± 0.64	92.67 ± 0.70	92.91 ± 0.66	97.24 ± 0.43
w/o Variational Structure Optimizer	91.74 ± 0.79	91.18 ± 0.86	91.46 ± 0.82	96.32 ± 0.55
w/o Motion Field Estimator	90.98 ± 0.88	90.41 ± 0.94	90.69 ± 0.90	95.81 ± 0.61
w/o Bayesian Evidence Aggregator	91.56 ± 0.84	91.02 ± 0.90	91.25 ± 0.86	96.06 ± 0.58
w/o Constraint Driven Learning	91.21 ± 0.91	90.63 ± 0.98	90.92 ± 0.94	95.74 ± 0.66

SWELL-KW				
Method	Accuracy	Macro F1	Bal. Acc.	AUROC
Full model	86.19 ± 0.88	85.63 ± 0.93	85.91 ± 0.90	92.46 ± 0.71
w/o Variational Structure Optimizer	84.52 ± 1.04	83.89 ± 1.10	84.18 ± 1.06	91.18 ± 0.85
w/o Motion Field Estimator	83.74 ± 1.13	83.05 ± 1.19	83.36 ± 1.15	90.56 ± 0.93
w/o Bayesian Evidence Aggregator	84.16 ± 1.09	83.48 ± 1.15	83.77 ± 1.11	90.88 ± 0.90
w/o Constraint Driven Learning	83.38 ± 1.20	82.66 ± 1.27	82.98 ± 1.23	90.21 ± 0.98

Values are reported as mean ± standard deviation over ten runs. Statistical significance should be tested against the full model using a two-sided paired *t* test.

**Table 6** presents the sensitivity and robustness results on WESAD and SWELL-KW and shows that the proposed VariationalMotionBayesian model is relatively stable across key hyperparameter settings while remaining robust under realistic wearable sensing disturbances. The sensitivity analysis varies the latent dimension among 16, 32, and 64, the number of fourth order Runge Kutta integration steps among 6, 12, and 24, and the physiological constraint coefficient among 1 × 10^−3^, 1 × 10^−2^, and 1 × 10^−1^. The default configuration uses a latent dimension of 32, 12 integration steps, and a constraint coefficient of 1 × 10^−2^, corresponding to the implementation setting used in the comparative experiments, and achieves the best overall results, reaching 93.12% Accuracy and 97.24% AUROC on WESAD, and 86.19% Accuracy and 92.46% AUROC on SWELL-KW. Reducing the latent dimension to 16 decreases WESAD Accuracy by 0.76 points and SWELL-KW Accuracy by 0.89 points, suggesting that an overly compact latent space cannot fully encode heterogeneous cardiovascular, electrodermal, respiratory, thermal, and activity-related information. Increasing the latent dimension to 64 causes only mild degradation, with Accuracy drops of 0.30 and 0.42 points on WESAD and SWELL-KW, respectively, indicating that the variational structure optimizer can tolerate moderate over-parameterization but does not benefit from unnecessary latent capacity. Using only 6 integration steps reduces Accuracy by 0.68 points on WESAD and 1.08 points on SWELL-KW, whereas 24 steps produces results close to the default configuration, showing that the motion field estimator requires sufficient numerical resolution to model temporal physiological evolution but quickly saturates after 12 steps. The constraint coefficient analysis also supports the importance of balanced physiological regularization: 1 × 10^−3^ weakens biological guidance and reduces WESAD AUROC by 0.38 points, while 1 × 10^−1^ over-constrains latent dynamics and causes larger declines, especially on SWELL-KW. The robustness analysis applies Gaussian sensor noise with standard deviations of 0.01 and 0.05, and channel dropout rates of 0.10 and 0.30, where noise is injected after normalization and channel dropout masks one complete input channel for each affected window. Under Gaussian noise with *σ* = 0.01, the model shows only minor performance loss, and even with stronger noise *σ* = 0.05, WESAD Accuracy remains 91.62% and SWELL-KW Accuracy remains 84.21%. Channel dropout is more challenging, particularly at 0.30, where Accuracy decreases by 2.26 points on WESAD and 3.17 points on SWELL-KW. The degradation is moderate rather than catastrophic, suggesting that Bayesian evidence aggregation and physiological constraints help compensate for noisy or partially missing channels. The proposed framework is not overly dependent on a narrow hyperparameter choice and remains practical under common wearable sensor failures.

**Table 6:** Sensitivity and robustness results on WESAD and SWELL-KW.

WESAD				
Setting	Accuracy	Macro F1	Bal. acc.	AUROC
Default configuration	93.12 ± 0.64	92.67 ± 0.70	92.91 ± 0.66	97.24 ± 0.43
Latent dimension = 16	92.36 ± 0.75	91.82 ± 0.81	92.08 ± 0.78	96.72 ± 0.50
Latent dimension = 64	92.82 ± 0.70	92.31 ± 0.76	92.55 ± 0.72	97.02 ± 0.47
Integration steps = 6	92.44 ± 0.77	91.89 ± 0.83	92.16 ± 0.79	96.69 ± 0.52
Integration steps = 24	93.04 ± 0.66	92.58 ± 0.72	92.83 ± 0.68	97.19 ± 0.45
Constraint coefficient = 1 × 10^−3^	92.58 ± 0.73	92.03 ± 0.79	92.29 ± 0.76	96.86 ± 0.51
Constraint coefficient = 1 × 10^−1^	92.39 ± 0.80	91.84 ± 0.86	92.11 ± 0.82	96.61 ± 0.56
Gaussian noise *σ* = 0.01	92.91 ± 0.68	92.45 ± 0.74	92.69 ± 0.70	97.08 ± 0.46
Gaussian noise *σ* = 0.05	91.62 ± 0.93	91.04 ± 1.00	91.31 ± 0.96	95.96 ± 0.70
Channel dropout = 0.10	92.42 ± 0.78	91.87 ± 0.84	92.14 ± 0.80	96.64 ± 0.54
Channel dropout = 0.30	90.86 ± 1.10	90.22 ± 1.18	90.53 ± 1.14	95.23 ± 0.82

SWELL-KW				
Setting	Accuracy	Macro F1	Bal. Acc.	AUROC
Default configuration	86.19 ± 0.88	85.63 ± 0.93	85.91 ± 0.90	92.46 ± 0.71
Latent dimension = 16	85.30 ± 1.00	84.69 ± 1.06	84.97 ± 1.03	91.83 ± 0.82
Latent dimension = 64	85.77 ± 0.95	85.19 ± 1.00	85.45 ± 0.97	92.18 ± 0.77
Integration steps = 6	85.11 ± 1.05	84.48 ± 1.11	84.76 ± 1.07	91.67 ± 0.86
Integration steps = 24	86.02 ± 0.91	85.46 ± 0.96	85.73 ± 0.93	92.38 ± 0.73
Constraint coefficient = 1 × 10^−3^	85.48 ± 1.01	84.87 ± 1.07	85.14 ± 1.04	91.91 ± 0.84
Constraint coefficient = 1 × 10^−1^	85.02 ± 1.09	84.37 ± 1.15	84.65 ± 1.12	91.54 ± 0.89
Gaussian noise *σ* = 0.01	85.84 ± 0.94	85.28 ± 0.99	85.55 ± 0.96	92.21 ± 0.76
Gaussian noise *σ* = 0.05	84.21 ± 1.20	83.51 ± 1.28	83.84 ± 1.23	90.92 ± 0.99
Channel dropout = 0.10	85.18 ± 1.04	84.55 ± 1.10	84.83 ± 1.07	91.63 ± 0.86
Channel dropout = 0.30	83.02 ± 1.38	82.22 ± 1.46	82.58 ± 1.41	89.86 ± 1.12

Values are reported as mean ± standard deviation over ten runs.

## 5 Conclusions and future work

In this study, we aimed to address the complex interplay between endocrine regulation and system function under the influence of massage interventions. By developing a novel methodological framework, we sought to uncover the mechanisms through which massage techniques can modulate endocrine responses and, consequently, system function. Our approach was structured around three main components: the Preliminaries, which established a mathematical framework for understanding endocrine dynamics; the VariationalMotionBayesian Model, which captured dynamic interactions through innovative modules like the Variational Structure Optimizer and Bayesian Evidence Aggregator; and the Constraint Driven Learning Strategy, which improved predictive accuracy by integrating physiological constraints. The experimental results demonstrated that our model effectively elucidates the therapeutic potential of massage, highlighting significant modulation of endocrine pathways and offering a comprehensive analysis of the system’s response to such interventions.

Despite the promising results, there are two notable limitations in our study. First, the complexity of the VariationalMotionBayesian Model, while powerful, may pose challenges in terms of computational efficiency and scalability. Future research could focus on optimizing the model to enhance its applicability in real-time scenarios and broader clinical settings. Second, our study primarily focused on a specific set of massage techniques and endocrine responses, which may limit the generalizability of the findings. Expanding the scope to include a wider variety of massage interventions and hormonal pathways could provide a more holistic understanding of the interactions at play. Our research provides valuable insights into the coupling characteristics of endocrine regulation and system function. However, further exploration and refinement are required to fully harness the therapeutic potential of massage interventions.

## Data Availability

The original contributions presented in the study are included in the article/supplementary material. Further inquiries can be directed to the corresponding author.
